# Prevalence and severity of Bertolottis syndrome in Malaysia: A common under diagnosis

**DOI:** 10.51866/oa.64

**Published:** 2022-10-18

**Authors:** Mohamad Faiz Noorman, Ahmad Anuar Sofian, Mohd Khairuddin Kandar, Ashraf Hakim Ab Halim, Mohd Hezery Harun, Fadzrul Abbas Mohamed Ramlee, Fahrudin Che Hamzah, Ezamin Abdul Rahim

**Affiliations:** 1 MD (UKM), Master in Orthopaedic Surgery (UPM), Department of Orthopaedic, Faculty of Medicine, Universiti Teknologi MARA (UiTM), Jalan Hospital, Sungai Buloh Campus, Selangor, Malaysia. Email: faiznoorman@gmail.com; 2 MBBS (CUCMS), Master in Orthopaedic Surgery (UPM), Department of Orthopaedic, Faculty of Medicine, Universiti Teknologi MARA (UiTM), Jalan Hospital, Sungai Buloh Campus, Selangor, Malaysia.; 3 MD (UKM), Master in Orthopaedic Surgery (UKM), Avisena Specialist Hospital, Jalan Ikhtisas, Seksyen 14, Shah Alam, Selangor, Malaysia.; 4 MD (UKM), Master in Orthopaedic Surgery (UKM), Department of Orthopaedic, Faculty of Medicine and Health Sciences, Universiti Putra Malaysia, Serdang, Selangor, Malaysia.; 5 (MD (USM), Doctor of Orthopaedic & Traumatology (UKM), Department of Orthopaedic, Faculty of Medicine and Health Sciences, Universiti Putra Malaysia, Serdang, Selangor, Malaysia.; 6 MBBS (Ireland), Doctor of Orthopaedic & Traumatology (UKM), Department of Orthopaedic, Faculty of Medicine and Health Sciences, Universiti Putra Malaysia, Serdang, Selangor, Malaysia.; 7 MD (UKM), Master in Orthopaedic Surgery (UKM), Department of Orthopaedic, Faculty of Medicine and Health, Sciences, Universiti Putra Malaysia, Serdang, Selangor, Malaysia.; 8 MD (UKM), MMed (Radiology)(UKM), Department of Radiology, Faculty of Medicine and Health Sciences, Universiti Putra Malaysia, Serdang, Selangor, Malaysia.

**Keywords:** Bertolotti's syndrome, Low back pain, Lumbosacral transitional vertebrae, Underdiagnosis

## Abstract

**Introduction:**

Bertolotti’s syndrome (BS) is defined as the presence of low back pain (LBP), radiculopathy or both with a dysplastic transverse process (TP) of the fifth lumbar vertebra that is articulated or fused with the sacral base or iliac crest. This study aimed to investigate the prevalence and severity of BS to promote awareness of this disease.

**Method:**

A retrospective review of anteroposterior lumbosacral plain radiographs was conducted between 1 January and 31 December 2017. Patients were recruited via systematic randomised sampling and were then interviewed and examined. The severity of BS was measured objectively using the numerical pain rating scale (NPRS) and Oswestry disability questionnaire (ODQ). Data were analysed using IBM SPSS for Windows version 22.

**Results:**

The prevalence of BS was 9.6% (16/166). Age significantly affected the severity of BS. The older and younger groups had a mean ODQ score of 42.86% and 24.08%, respectively (P=0.006). There was no significant relationship found between the prevalence of BS and age (P=0.126). Only one patient was diagnosed with BS during medical consultation. The mean NPRS score was 5.5. The majority of the BS cases were of moderate severity (43.8%), followed by those of minimal severity (31.2%) and severe disability (25%).

**Conclusion:**

Early diagnosis of BS and orthopaedic referral are crucial to halt its progression. BS should be considered in patients presenting with LBP during assessments of lumbosacral radiographs.

## Introduction

Bertolotti’s syndrome (BS) is named after Mario Bertolotti who described this condition in 1917. Lumbosacral transitional vertebrae (LSTV) are a congenitally morphological spinal variation that spans a spectrum from a dysplastic transverse process (TP) of the fifth (L5) lumbar vertebra to partial/complete fusion between the TP of L5 and sacral base or iliac crest.^1-5^ LSTV associated with low back pain (LBP), radiculopathy or both is defined as BS.

The exact cause of LBP in patients with BS remains uncertain, although a few theories mainly attributed to arthritic changes and disc degeneration have been postulated.^1^ Specifically, reference has been made to the fact that the disc above the transitional vertebra appears to be at risk of increased degenerative changes, while the disc below appears to be protected.^3,6^ These findings are also supported by Aihara et al., who proposed that this pathophysiology is attributed to hypermobility and abnormal torque of the intervertebral disc space immediately above the transitional vertebra, which appears to be more concentrated than do other levels.^6^ Once disc degeneration occurs, further mechanical irritation of the nerve root by the degenerated disc or pseudo-joint may lead to radicular symptoms.^7^

The prevalence of BS in the general population varies in the literature. It has been reported to be between 4.6% and 7% and reach up to 11.4% in patients under the age of 30 years.^2,3^ Further, Castellvi et al. found a high prevalence of lumbosacral anomalies of 30%.^1^ Although Tini et al. suggested that LSTV was not associated with LBP, other studies indicated an association of LSTV with LBP and buttock pain.^3-5,7-9^

Currently, data on the prevalence, severity and underdiagnosis rate of BS in Malaysia are scarce. In the country, LBP is a common presentation in daily clinical practice and commonly being assessed initially by local medical practitioners ranging from primary care to emergency doctors who subsequently offer orthopaedic referral. However, not many medical practitioners are aware of this condition, which leads to delay in its diagnosis and treatment. In Malaysia, Manmohan et al. reported the case of a 20-year-old lady who experienced marked back pain for 8 years for which she had visited general practitioners (GPs) regularly and underwent various advanced imaging studies, including lumbosacral magnetic resonance imaging (MRI) (thrice), cervical spine MRI (once), lumbar spine computed tomography (once), plain lumbosacral spine radiography (LSR; eight times) and nerve conduction study (once) prior to the diagnosis of BS.^10^

Accordingly, this study is expected to add to the existing body of literature information promoting the awareness of this disease, earlier diagnosis and orthopaedic referral if clinically indicated. This would consequently help reduce the economic burden by reducing unnecessary tests and multiple visits to GPs and emergency departments. Early diagnosis with timely management provides relief of acute pain and helps prevent chronic LBP along with its complications. Symptomatic patients whose conservative management failed may be initially treated with local steroid and anaesthetic agent injections.^2,4,5^ Therefore, this study aimed to determine the prevalence, severity and underdiagnosis rate of BS in Malaysia.

## Materials and methods

### Study design and population

The study was performed in Hospital Serdang, Malaysia. Ethical approval was provided by the Medical Research and Ethics Committee, Ministry of Health Malaysia prior to the initiation of the study. Six hundred anteroposterior (AP) plain LSRs that were taken in 2017 were selected via purposive sampling from 1 January to 31 December 2017. We selected 50 LSRs of the patients for each month, which totalled up to 600 LSRs over 12 months.

In purposive sampling, the LSRs of the patients were arranged according to the date from the first day of the month until the end of the month between January and December 2017. Once arranged, the LSRs were selected and reviewed to select the first 50 radiographs for each month up to a total of 600 radiographs for 12 months. Only the LSRs of the patients who fulfilled the inclusion criteria were selected. Radiographs were reviewed using the Zero Footprint medical image viewer software, which is the default radiographic viewer in Hospital Serdang.

**Figure 1:**
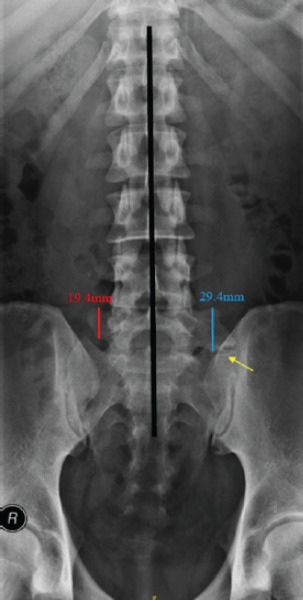
All spinous processes in the straight line and the pedicle distance to the spinous process on each side were similar, showing a true AP* view of the lumbosacral plain radiograph. L5 was set in a cephalad-to-caudal manner where the 12th rib was the marker and corresponded to T12. Type I LSTV was noted on the right transverse process of L5, measured 19.4 mm and dysplastic (red line). Type II LSTV was observed on the left side and measured 29.4 mm (blue line) with evidence of pseudo-arthrosis between the transverse process of L5 and sacrum (yellow arrow). *AP: Anteroposterior

The inclusion criteria were Malaysian nationality, age between 18 and 60 years and true AP view of the LSR of the patients who visited Hospital Serdang in 2017. A true AP view of the lumbosacral radiograph was defined as the view showing all spinous processes in a straight line, in which the pedicle distance to the spinous process on each side was similar (**Figure 1**). Meanwhile, the exclusion criteria were pregnancy, known history of any lumbosacral spine fractures and spinal disc pathology, including degenerative disc disease and prolapse intervertebral disc, and plain radiographic evidence of the following: previous lumbosacral surgery, lumbosacral spine fracture, spinal tumours, other congenital lumbosacral malformation and spinal infections.

**Figure 2:**
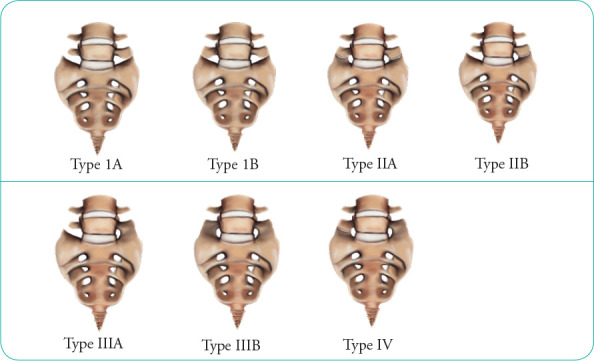
Illustration of the Castellvi classification

Radiological assessment was performed using the AP-view LSRs to accurately determine the size of the TP of L5, which was measured between the uppermost and lowermost points (**Figure 1**). Subsequently, Castellvi classification was used to describe the morphologic appearance of LSTV (**Figure 2**).^1^ The four types in this classification were then further subclassified into unilateral (a) or bilateral (b) involvement (**Figure 2**): Type I: Dysplastic TP (>19 mm); Type II: Incomplete lumbarisation/sacralisation (pseudo-articulation); Type III: Complete lumbarisation/sacralisation (bony union) between the TP and sacrum; Type IV: Mixture of types II and III on each side. In our study, we used the simplified classification by Nardo et al. where the cases were not further classified into bilateral or unilateral to minimise the number of categories.^8^

As no comparable previous local studies could be found, the most current study available on the prevalence of the BS in the general population by Jancuska et al., which reported a mean of 12.3%, was tailored for our use.^5^ A minimum sample size of 166 patients was required for our study. The registration numbers of 600 patients were recorded in a Microsoft Office Excel spreadsheet, which was then used to generate unique identification numbers. Systematic randomised sampling was performed using Microsoft Office Excel according to the patients’ unique identification number, which yielded 166 eligible patients for inclusion.

Of the 166 patients, only those who had LSTV were interviewed and examined. No appointments were made for those without LSTV. This was because a clinical history of LBP, radicular symptoms or both with the finding of LSTV must be present to establish a final diagnosis of BS.^1-4^

### Study procedure

A retrospective review of electronic medical records (EMRs) was performed to collect the demographic data, including age, sex and ethnicity, of the 166 patients. We identified 16 patients with LSTV, who were then interviewed and examined. Informed consent was obtained, followed by focused history-taking, clinical examination and questionnaire completion. The examinations conducted included localisation of low back tenderness and routine neurological lower limb examination. Back tenderness localisation was divided into lumbar, buttock, radiculopathy and others where specification was required. Lower limb neurological examinations followed the American Spinal Injury Association chart of key muscles and key sensory points.^11^

The pain severity was measured using three scales, one for pain during the entire present episode, while the other two were for usual pain during the previous week and during the last episode (when there was no episode of back pain in the past week). In all cases, the pain scale used was the 11-point numerical pain rating scale (NPRS) anchored at one end by the label ‘no pain’ and at the opposite end by ‘worst pain possible’. The question asked was as follows: We realise that there have been ‘good’ and ‘bad’ days, but, on average, how would you rate the severity of your back pain: during the entire time you have had it, during the past week and during the last episode (if there was no episode of back pain in the past week)? This format of questioning has been successfully used in previous studies and is a well-validated method of measuring a patients’ pain experience.^12-14^

The patients were then asked to answer the Oswestry disability questionnaire (ODQ) with our guidance to specifically assess the severity of LBP. This questionnaire comprises 10 questions. Each question has a total possible score of 5. When the first statement is marked, the score is 0; when the last statement is marked, then the score is 5. The final scores are obtained by dividing the total scores with the total possible scores and multiplying them by 100. This is also well validated in the literature.^12,15^ The scores are interpreted as follows: 0%-20%: minimal disability, 21%-40%: moderate disability, 41%-60%: severe disability, 61%-80%: crippled and 81%-100%: either bed-bound status or exaggerated symptoms.

The weight and height were measured to calculate the body mass index (BMI) defined as weight in kilograms divided by height in metres squared. The BMI was measured and classified in accordance with the World Health Organization guidelines into underweight (<18.50 kg/m^2^), normal (18.50-24.99 kg/m^2^) and overweight (≥25.00 kg/m^2^).^16^

### Quality control

The measurements were conducted by two independent evaluators on the same occasion. Both evaluators had more than 5 years of experience as an orthopaedic surgeon and a radiologist. This was ensured to achieve an accurate measurement upon radiological assessment.

### Data analysis

Standard descriptive data were expressed as frequencies (percentages) for all qualitative variables. The chi-square test or Yates continuity correction was used for categorical data analysis and Student’s t-test for continuous data analysis. Statistical significance was set at P-values of <0.05. The IBM SPSS Statistics for Windows, Version 22.0 (Armonk, New York: IBM Corp) was used to conduct the analyses.

## Results

A total of 166 patients (age: 18-60 years) who satisfied the inclusion criteria were selected on the basis of the reviewed LSRs of the 600 patients. Of them, 16 were diagnosed with BS (9.6%). Seven out of these sixteen patients had Castellvi type I (43.8%); six, type II (37.5%); and three, type III (18.7%). Fourteen patients presented with LBP one patient with radiculopathy and one patient with both LBP and radiculopathy. The mean duration of symptoms was 6.81 years (standard deviation [SD]: 5.167), with no significant association to the severity of BS measured using the ODQ (P=0.690).

Fifteen patients had previous medical consultations with primary care and emergency doctors. The common diagnoses made were mechanical back pain (n=7), prolapsed intervertebral disc (n=5) and degenerative lumbar disease (n=2). Only one patient was offered orthopaedic referral, and appropriate treatment was then initiated. One patient did not seek any medical attention; the LSR was obtained owing to other reasons. No significant differences were found between BS and age, sex and ethnicity (**Table 1**).

**Table 1:** Relationship between the demographic data and BS.

Variable	Diagnosis^†^		Total no.	P-value (χ^2^)	OR (95% CI)
	BS	Normal			
**Age**					
≤30 years	9 (14.1)	55 (85.9)	64	0.126 (2.341)	2.22 (0.783-6.296)
>30 years	7 (6.9)	95 (93.1)	102		
**Sex**					
Male	8 (9.3)	78 (90.7)	86	0.879 (0.023)	0.923 (0.329-2.588)
Female	8 (10)	72 (90)	80		
**Ethnicity**					
Malay	14 (11.3)	110 (88.7)	124	0.349^*^ (0.877)	2.545 (0.554-11.698)
Non-Malay	2 (4.8)	40 (95.2)	42		

BS, Bertolotti’s syndrome; OR, odds ratio; CI, confidence interval.

* Derived using Yates continuity correction.

† Data are presented as numbers (%).

The severity of BS was measured using the NPRS score, which ranged from 2 to 8, with a mean score of 5.5 (entire time and last episode). Based on the ODQ score, five patients had minimal back pain; seven, moderate; and four, severe. The mean BMI was 29.59 (SD: 4.975; range: 22.86-38.05) kg/m^2^. Age was significantly associated with the severity of BS (**Table 2**).

**Table 2:** Relationship of the demographic data and BMI with the severity of Bertolotti’s syndrome.

Variable	Mean ODQ score, % (SD)	Total no.	P-value^*^
**Age**			
<30 years	24.078 (12.239)	4	0.006 (3.260)
>30 years	42.857 (10.254)	12	
**Sex**			
Male	30.863 (17.465)	8	0.710 (0.380)
Female	33-726 (12.246)	8	
**Ethnicity**			
Malay	31.336 (15.475)	14	0.508 (0.680)
Non-Malay	39(1.414)	2	
**BMI**			
Normal	30.50 (14.640)	4	0.710 (0.380)
Overweight	32.892 (15.249)	12	
**Mean NPRS score during the entire time (SD)**			
**Sex**			
Male	5.250 (2.121)	8	0.544 (0.632)
Female	5.750 (0.707)	8	
**Ethnicity**			
Malay	5-357 (1.598)	14	0.346 (0.974)
Non-Malay	6.5 (0.707)	2	
**BMI**			
Normal	5.750 (0.957)	4	0.723 (0.362)
Overweight	5.417(1.730)	12	
**ODQ finding**			
Minimal	4(1.826)	4	0.019 (2.646)
Moderate to severe	6 (1.128)	12	
**Mean NPRS score during the last episode (SD)**			
**Sex**			
Male	5-375 (2.387)	8	0.703 (0.392)
Female	5.750 (1.282)	8	
**Ethnicity**			
Malay	5.357 (1.865)	14	0.256 (1.183)
Non-Malay	7.0 (1.414)	2	
**BMI**			
Normal	5.250 (1.259)	4	0.712(0.377)
Overweight	5.667 (2.060)	12	
**ODQ finding**			
Minimal	3.75 (2.062)	4	0.018 (2.670)
Moderate to severe	6.17(1.403)	12	

ODQ, Oswestry disability questionnaire; NPRS, numerical pain rating scale; BMI, Body Mass Index; SD, standard deviation.

* Derived from Student’s t-test

## Discussion

In the literature, the prevalence of BS ranges between 4.0% and 37%.^1,3,4,6-8^ The variability and wide range in the prevalence may be attributed to varying factors, including narrow age span, inclusion of patients without back pain but with other diseases or biased selection of patients.^5^ As previously described, the prevalence of BS was higher in studies that included patients with LBP and lower in community-based studies.^8^ Although our patients were from a single hospital, the prevalence of BS in this study (9.6%) was within the range of previous reports. Additionally, no significant difference was observed between the male (9.3%) and female (10%) groups, which is comparable to earlier findings, although some showed a higher prevalence of BS in men.^7,17^ The prevalence of BS was not significantly associated with age (P=0.126), also similar to previous findings.^3^ To our knowledge, this is the first study of its kind in Malaysia; it showed similar results with the study by Nardo et al. that there was no significant relationship between BS and ethnic groups (P=0.363).^8^

The simple NPRS used in this study could represent the severity and disability of LBP as it was significantly related to the detailed ODQ score. This indicates that this simple, more rapid and accurate method should be utilised by local medical practitioners in assessing the severity of LBP. Age was also found to be significantly associated with the severity of BS. In this study, the patients aged >30 years had a higher mean ODQ score (42.9%) than those aged <30 years (24.1%) (P=0.006). This finding is also similar with the findings by Peterson et al. that older patients reported more disability than did younger patients.^12^

To the best of our knowledge, no study has shown underdiagnoses of patients with BS in Malaysia. In this study, 14 of 15 patients were not diagnosed with BS during their medical consultations. This shows the importance of early detection of BS, so that timely intervention of the condition could be provided. Early detection can also help prevent worsening of LBP which was significantly observed in the older patients in this study. It can be achieved by having a high index of suspicion upon clinical assessment of patients with LBP inclusive of clinical history, physical examination and appropriate LSR assessment to identify any radiological anomalies, including LSTV. In addition, LBP is one of the main reasons for needing medical assistance, and 80% of adults seek help at some stage in life. In the USA, approximately 15%-20% of the population is affected by LBP and an estimated USD 50 billion is the annual cost for diagnosing and treating LBP.^3^ A proper radiological assessment of plain LSRs can prevent medical practitioners from performing unwanted advanced imaging studies, which may subsequently reduce the economic burden.

Herein, all patients with LSTV were diagnosed with BS. This finding is contradicted by a previous literature where only 57.6% of patients with LSTV were diagnosed with BS.^8^ One possible explanation may be the difference in the sample size used. Nevertheless, our study also has some limitations. It was conducted in a single health care centre and included a relatively small number of patients. We used systematic randomised sampling to lessen the effect of this limitation and to ascertain that this study may represent our local community.

## Conclusion

The prevalence of BS among patients in Malaysia was 9.6%. The older patients were more likely to present moderate-to-severe symptoms than the younger patients. All patients with LSTV were diagnosed with BS. Of the 15 patients with BS, 14 were undiagnosed upon medical consultation, making it an utmost necessity to include BS as part of the differential diagnosis of LBP
